# Supplementary material for: Synthetic Adrenocorticotropic Peptides Modulate the Expression Pattern of Immune Genes in Rat Brain following the Early Post-Stroke Period
Source: Genes (Basel). 2023 Jun 30;14(7):1382. doi: 10.3390/genes14071382 (PMC10379992; doi:10.3390/genes14071382)
Supplement: Supplementary file 1 [file genes-14-01382-s001.zip › Supplementary Figure S1.pptx]

## Slide 1
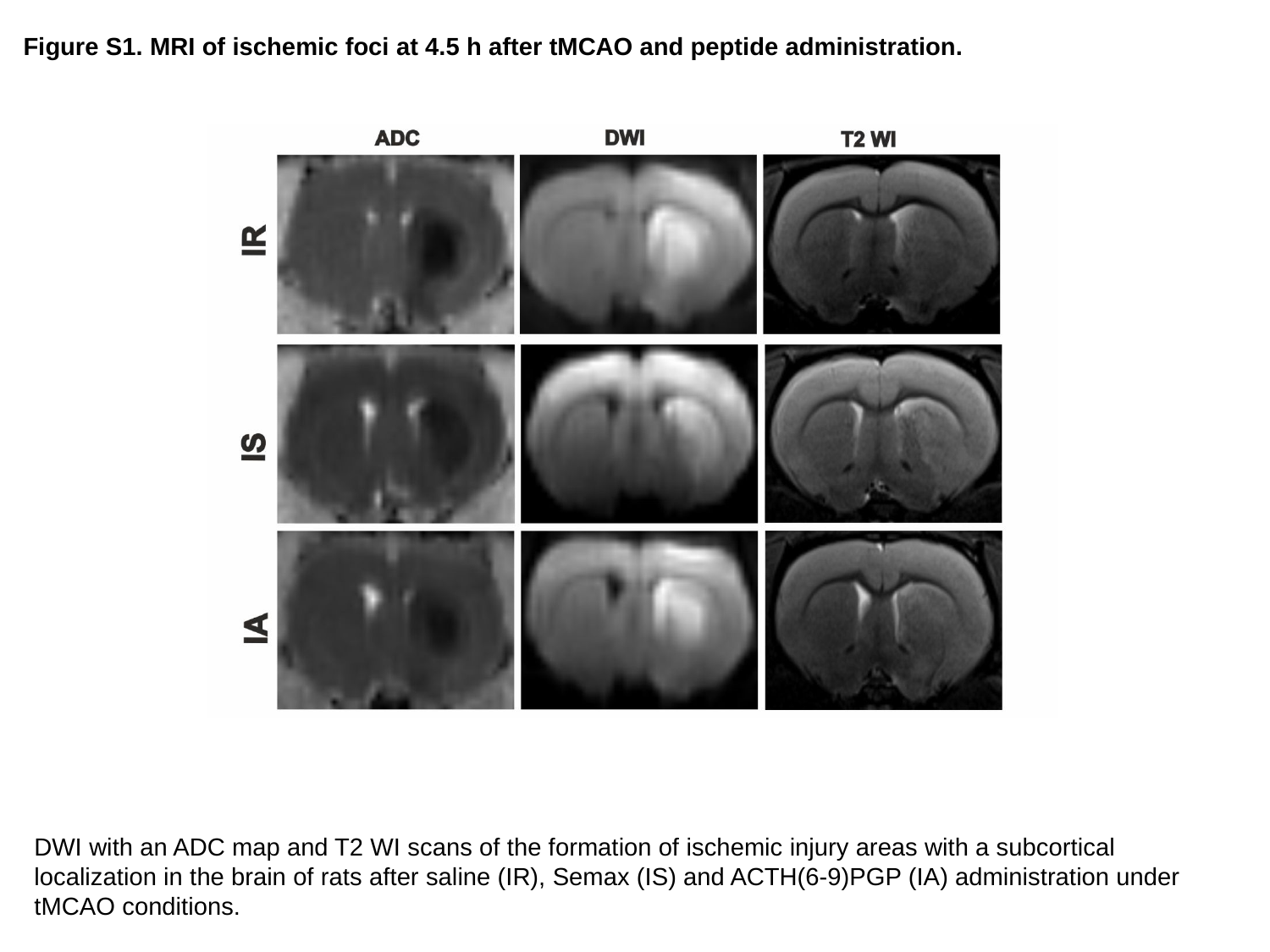

# Figure S1. MRI of ischemic foci at 4.5 h after tMCAO and peptide administration.
DWI with an ADC map and T2 WI scans of the formation of ischemic injury areas with a subcortical localization in the brain of rats after saline (IR), Semax (IS) and ACTH(6-9)PGP (IA) administration under tMCAO conditions.
