# Supplementary material for: Synthetic Adrenocorticotropic Peptides Modulate the Expression Pattern of Immune Genes in Rat Brain following the Early Post-Stroke Period
Source: Genes (Basel). 2023 Jun 30;14(7):1382. doi: 10.3390/genes14071382 (PMC10379992; doi:10.3390/genes14071382)
Supplement: Supplementary file 1 [file genes-14-01382-s001.zip › Supplementary Figure S2.pptx]

## Slide 1
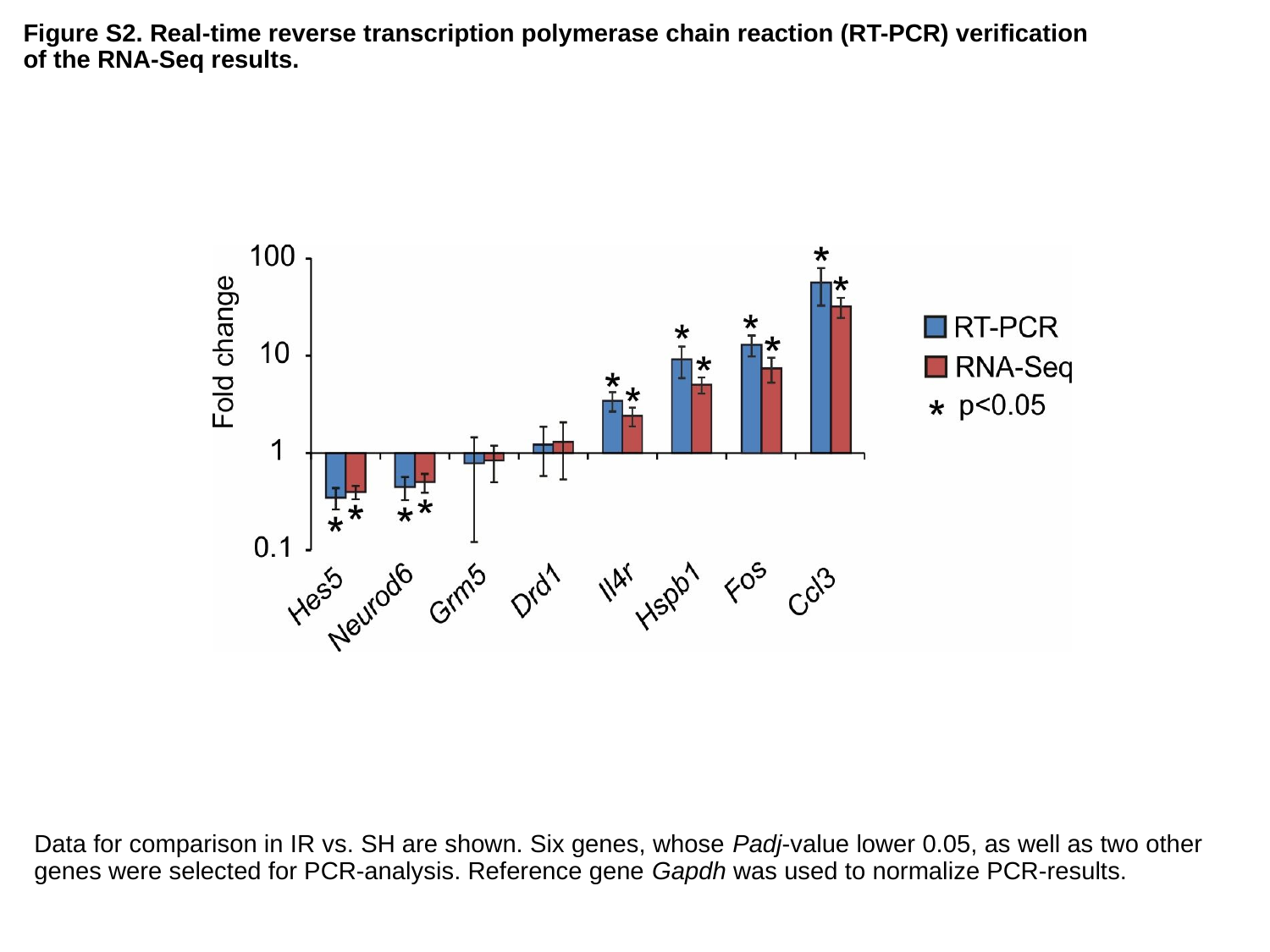

# Figure S2. Real-time reverse transcription polymerase chain reaction (RT-PCR) verification of the RNA-Seq results.
Data for comparison in IR vs. SH are shown. Six genes, whose Padj-value lower 0.05, as well as two other genes were selected for PCR-analysis. Reference gene Gapdh was used to normalize PCR-results.
