# Supplementary material for: Synthetic Adrenocorticotropic Peptides Modulate the Expression Pattern of Immune Genes in Rat Brain following the Early Post-Stroke Period
Source: Genes (Basel). 2023 Jun 30;14(7):1382. doi: 10.3390/genes14071382 (PMC10379992; doi:10.3390/genes14071382)
Supplement: Supplementary file 1 [file genes-14-01382-s001.zip › Supplementary Figure S3.pptx]

## Slide 1
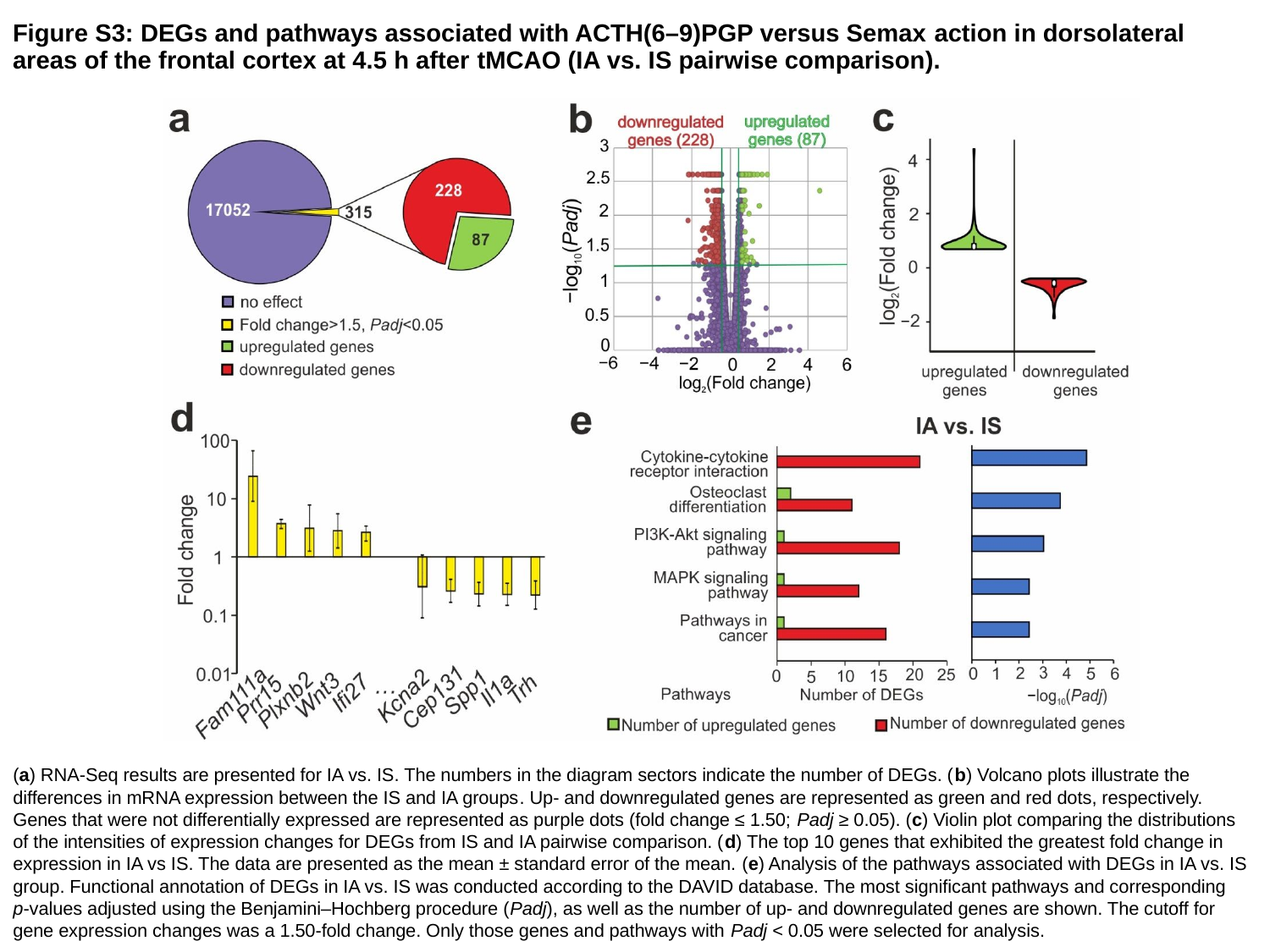

# Figure S3: DEGs and pathways associated with ACTH(6–9)PGP versus Semax action in dorsolateral areas of the frontal cortex at 4.5 h after tMCAO (IA vs. IS pairwise comparison).
(a) RNA-Seq results are presented for IA vs. IS. The numbers in the diagram sectors indicate the number of DEGs. (b) Volcano plots illustrate the differences in mRNA expression between the IS and IA groups. Up- and downregulated genes are represented as green and red dots, respectively. Genes that were not differentially expressed are represented as purple dots (fold change ≤ 1.50; Padj ≥ 0.05). (c) Violin plot comparing the distributions of the intensities of expression changes for DEGs from IS and IA pairwise comparison. (d) The top 10 genes that exhibited the greatest fold change in expression in IA vs IS. The data are presented as the mean ± standard error of the mean. (e) Analysis of the pathways associated with DEGs in IA vs. IS group. Functional annotation of DEGs in IA vs. IS was conducted according to the DAVID database. The most significant pathways and corresponding  p-values adjusted using the Benjamini–Hochberg procedure (Padj), as well as the number of up- and downregulated genes are shown. The cutoff for gene expression changes was a 1.50-fold change. Only those genes and pathways with Padj < 0.05 were selected for analysis.
